# Supporting HIV prevention and reproductive goals in an HIV-endemic setting: taking safer conception services from policy to practice in South Africa

**DOI:** 10.7448/IAS.20.2.21271

**Published:** 2017-02-20

**Authors:** Natasha ECG Davies, Lynn T Matthews, Tamaryn L Crankshaw, Di Cooper, Sheree R Schwartz

**Affiliations:** ^a^ Wits Reproductive Health and HIV Institute, University of the Witwatersrand, Johannesburg, South Africa; ^b^ Massachusetts General Hospital, Global Health, Division of Infectious Disease, Boston, MA, USA; ^c^ Health Economics and HIV and AIDS Research Division (HEARD), University of KwaZulu-Natal, Durban, South Africa; ^d^ School of Public Health, University of the Western Cape, Cape Town, South Africa; ^e^ Department of Epidemiology, Johns Hopkins Bloomberg School of Public Health, Baltimore, MD, USA

**Keywords:** HIV prevention, HIV treatment, safer conception, reproductive rights, South Africa

## Abstract

**Introduction**: Safer conception care encompasses HIV care, treatment and prevention for persons living with HIV and their partners who desire children. In 2012, South Africa endorsed a progressive safer conception policy supporting HIV-affected persons to safely meet reproductive goals. However, aside from select research-supported clinics, widespread implementation has not occurred. Using South Africa as a case study, we identify key obstacles to policy implementation and offer recommendations to catalyse expansion of these services throughout South Africa and further afield.

**Discussion**: Four key implementation barriers were identified by combining authors’ safer conception service delivery experiences with available literature. First, strategic implementation frameworks stipulating where, and by whom, safer conception services should be provided are needed. Integrating safer conception services into universal test-and-treat (UTT) and elimination-of-mother-to-child-transmission (eMTCT) priority programmes would support HIV testing, ART initiation and management, viral suppression and early antenatal/eMTCT care engagement goals, reducing horizontal and vertical transmissions. Embedding measurable safer conception targets into these priority programmes would ensure accountability for implementation progress. Second, facing an organizational clinic culture that often undermines clients’ reproductive rights, healthcare providers’ (HCP) positive experiences with eMTCT and enthusiasm for UTT provide opportunities to shift facility-level and individual attitudes in favour of safer conception provision. Third, safer conception guidelines have not been incorporated into HCP training. Combining safer conception with “test-and-treat” training would efficiently ensure that providers are better equipped to discuss clients’ reproductive goals and support safer conception practices. Lastly, HIV-affected couples remain largely unaware of safer conception strategies. HIV-affected populations need to be mobilized to engage with safer conception options alongside other HIV-related healthcare services.

**Conclusion**: Key barriers to widespread safer conception service provision in South Africa include poor translation of policy into practical and measurable implementation plans, inadequate training and limited community engagement. South Africa should leverage the momentum and accountability associated with high priority UTT and eMTCT programmes to reinvigorate implementation efforts by incorporating safer conception into implementation and monitoring frameworks and associated HCP training and community engagement activities. South Africa’s experiences should be used to inform policy development and implementation processes in other HIV high-burden countries.

## Introduction

Safer conception care encompasses HIV care, treatment and prevention for individuals and couples desiring a child, in which one or both partners are living with HIV [1]. Safer conception services employ proven methods to prevent HIV transmission while supporting the reproductive rights of people living with or affected by HIV [2]. In high HIV prevalence settings, such services may reduce adult and infant HIV incidence by promoting HIV testing, antiretroviral therapy (ART) initiation and viral load suppression, whilst also improving clinical HIV outcomes including maternal and infant morbidity and mortality [3]. Although preconception care may positively impact broader maternal and child health outcomes, for the purposes of this commentary, the target audience for safer conception services includes any person living with HIV, or at risk of HIV acquisition, who desires a child, regardless of whether they are aware of their own, or their partner’s, HIV status or engaged in healthcare services.

South Africa has been at the forefront of piloting safer conception services. The country’s Contraception and Fertility Planning Policy (2012) marked a paradigm shift from a contraception-focused family planning model, focused primarily on preventing unintended pregnancies, to one inclusive of safer conception services to support healthy, planned pregnancies [4]. This shift occurred in response to South Africa’s high HIV-burden amongst women of reproductive age, increasing evidence of high fertility intentions and pregnancy rates in this group [5–10] and persistently high HIV acquisition rates around the time of pregnancy [11]. Services to prevent unintended pregnancies and support safe pregnancy are seen as key to reducing new HIV infections in young women and achieving elimination-of-mother-to-child-transmission (eMTCT) goals [4,12].

To date, safer conception services have been piloted in Gauteng, KwaZulu-Natal and Western Cape provinces [13–17]. Two demonstration projects, currently underway in Johannesburg, along with a former hospital-based service in KwaZulu-Natal, provide “proof-of-concept” evidence that low-cost, low-technology safer conception strategies, similar to those showing success in Europe and North America [18–20], can be implemented in a resource-constrained, HIV-endemic setting [13,15,16,21].

The demonstration projects operate in urban primary healthcare facilities where 6000–8000 ART clients are managed monthly. In the past two years, each has provided safer conception services to over 300 couples – from which one or both partners attended. To date, over 50% of male partners have attended with their female partners. Services include HIV counselling and testing (HCT), ART and viral-load monitoring for all HIV-positive partners, pre-exposure prophylaxis for HIV-negative partners, syphilis testing and syndromic STI screening and treatment, referral for male medical circumcision, cervical cancer screening, education to identify peak fertile days and self-insemination if the male partner is HIV negative. Following counselling about the risks and benefits of each strategy, clients choose their preferred prevention methods. Early successes include male engagement in HIV care, addressing detectable viral loads among ART-established patients, linking pregnant women to early antenatal care and eMTCT services and empowering HIV-negative individuals to utilize HIV prevention methods while achieving their reproductive goals. These projects provide preliminary evidence for high user demand, nurse-driven model feasibility and safety – with no HIV transmissions observed [15,21]. Importantly, these South African services were designed to provide low-cost, low-technology, nurse-driven, primary healthcare-based interventions to test feasibility for expansion across South Africa and other similar settings.

**Figure 1.**
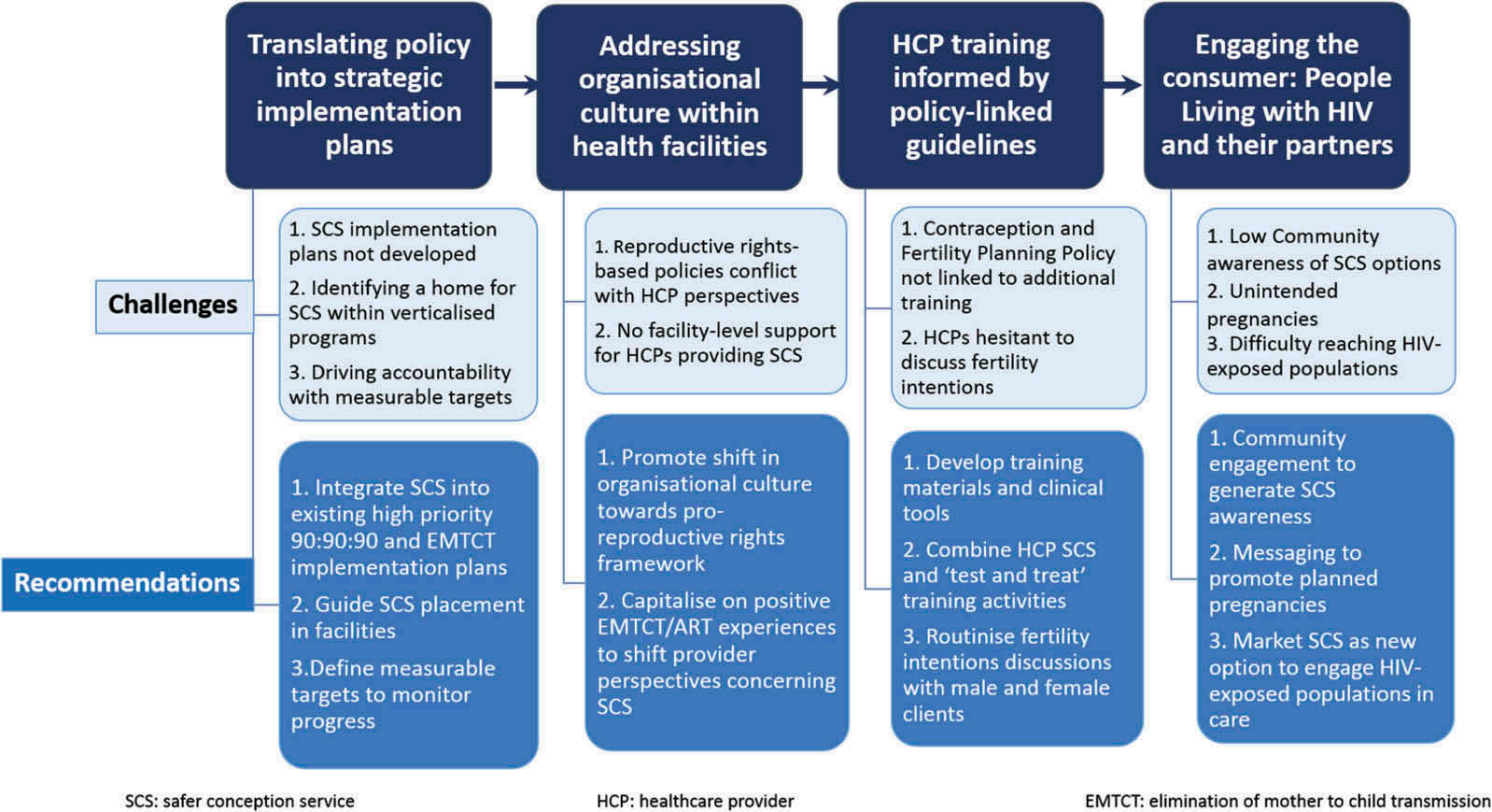
Safer conception service implementation: key gaps and recommendations. This figure summarizes four major gaps hindering South Africa’s translation of a supportive contraception and fertility planning policy into widespread safer conception service provision and proposes key recommendations to bridge these gaps.

Despite South Africa’s progressive policy and advocacy environment, evidence of demand and pilot project successes, broader integration of safer conception counselling into clinical care has not occurred. Moving from policy to action is complex, involving myriad, interdependent processes including effective leadership, strategic planning, data collection and analysis, stakeholder engagement and accountability, client and community awareness and knowledge and resource mobilization [22]. In this paper, we aim to examine how challenges in these areas impact South Africa’s progress towards translating its Contraception and Fertility Planning Policy into practice. Drawing on guidance from the USAID’s “moving policy to action” framework [22], we present South Africa as a case study, exploring four key gaps between policy and implementation (Figure 1): translating policy into strategic implementation plans, addressing organizational culture within healthcare facilities, provider awareness and knowledge and engaging people living with HIV (PLHIV), and their partners, who may benefit from safer conception services. We propose recommendations within each area to reinvigorate South Africa’s implementation efforts, thereby bridging the gap between aspirational policy goals and current realities. We hope this commentary will support development and implementation of safer conception policies across sub-Saharan Africa and beyond.

## Discussion

### Translating policy into strategic implementation plans

Following the launch of the Contraception and Fertility Planning Policy, numerous international and national guidelines have been released which bolster the argument for, and practically facilitate, broader safer conception service implementation; yet, implementation progress remains slow [17,20,23–25]. One key barrier to translation of policy into action has been a lack of clear implementation planning, including determining where safer conception services should be located, by whom they should be provided and according to what measurable targets. We argue that leveraging the potential of safer conception services to impact multiple high priority HIV programmes provides an opportunity to catalyse implementation. Safer conception services can simultaneously support universal test-and-treat (UTT) [23] associated UNAIDS 90:90:90 targets [13,26] and the eMTCT and broader HIV prevention goals of the Start Free, Stay Free, AIDS Free Initiative [27].

South Africa has committed to attaining 90% HCT uptake, 90% ART initiation and 90% viral load suppression, as per UNAIDS 90:90:90 targets [28]. In an effort to reach these goals, South Africa introduced UTT in September 2016 [29]. We propose that safer conception activities be incorporated into evolving UTT implementation plans and targets since this would represent an efficient alternative to a lengthy parallel process of developing a standalone safer conception framework. Integration of safer conception activities into the UTT plans finds congruence with 90:90:90 goals given the potential impact safer conception services may have on HCT and ART uptake, adherence and client retention, and viral suppression, particularly amongst men [15,30,31].

South Africa’s successful national eMTCT programme [32,33] presents another well-established prevention space into which safer conception services can be incorporated. Currently, South Africa’s eMTCT programme, like most global programmes, focuses on pillar 3 (preventing perinatal HIV transmission) and pillar 4 (care and support for HIV-positive mothers, their infants, partners and families) [34]. Scant attention has been paid to pillar 1 (HIV prevention in reproductive age women) and pillar 2 (reducing unintended pregnancies amongst women living with HIV) [34,35], though recognition of the importance of addressing these pillars in order to realize eMTCT goals is well established [30,34,35]. Safer conception care directly supports both pillars 1 and 2 by preventing new infections amongst women trying to conceive within serodiscordant partnerships and by using routine fertility intentions screening to identify clients eligible for safer conception and contraception services. Pillars 3 and 4 are also supported through earlier antenatal care booking with provision of quality care and earlier ART uptake by HIV-positive women, thus reducing perinatal transmission risks and supporting maternal health [15].

The crosscutting relevance of safer conception to 90:90:90 and eMTCT of HIV and syphilis goals provides opportunities to move safer conception service delivery forward. However, because HIV treatment, eMTCT and family planning services often exist within verticalized, separately funded, programmes [30], it becomes challenging to identify where to locate safer conception services and to whom responsibility for implementation should be assigned. Although safer conception services engage men who are motivated to protect their partner and/or child’s health, placing safer conception services within traditional family planning or eMTCT services risks excluding men, who are traditionally excluded from these services [31]. Women planning pregnancy may also avoid family planning clinics because they are solely perceived as contraception services. Similarly, placement within existing ART or HIV services risks alienating HIV-negative individuals and those reluctant to engage in HIV care. Integrating safer conception services into all of these programmes represents the most comprehensive answer but will be difficult to achieve. We therefore recommend embedding safer conception within HIV services, with family planning, STI, and antenatal and postnatal care providers receiving basic training to facilitate appropriate client referrals. A service delivery framework will need to clarify which providers and sites are responsible for safer conception service delivery, in order to engage relevant providers [36]. Communication strategies to inform communities of their availability will also be required. The framework can function as a practical guide for site level implementers (including a list of entry points for routine fertility intention screening), as a matrix of how to provide services in different types of healthcare facilities. This would incorporate the roles and responsibilities by levels of HCP, a summary of services and counselling strategies offered, a monitoring and evaluation framework with measurable targets, community mobilization activities and supply management tips, clinical support tools and additional training resources.

### Addressing organizational culture within health facilities

Policy does not exist in a vacuum. Implementation relies on a well-developed implementation framework and a supportive organizational culture [37]. South Africa’s commitment to reproductive rights is evidenced by its comprehensive, progressive sexual and reproductive health policies and laws [38]. However, the health system relies on a diversity of HCPs – doctors, nurses, lay counsellors, community-based health workers – each cadre operating within the context of differing social norms, prior training and medical culture [39]. An entrenched, stigmatizing or judgemental organizational culture can overwhelm positive attitudes of individual HCPs, leaving them unsupported and reinforcing negative attitudes, thus shaping provider behaviour [40]. Within this context, tensions arise between the policy-mandated rights-based approach to patient-centred care and HCPs’ own clinical perspectives, moral judgements and priorities, sometimes hampering client access to services, including safer conception care [16,41]. Encouragingly, provider attitudes towards PLHIV who desire children are rapidly evolving. Earlier research suggested that providers tended to focus on negative outcomes of childbearing for PLHIV, creating reluctance to advise condomless sex or other safer conception methods for PLHIV and their partners [6,42–44]. However, recent studies indicate that providers increasingly empathize with reproductive goals of PLHIV, recognize reduced perinatal transmissions following eMTCT interventions, acknowledge the need for safer conception training and care and show greater awareness of safer conception methods [45–50]. In order to cement this shift, widespread promotion of a reproductive-rights based patient care approach is required. For example, the strong leadership and accountability built into the eMTCT programme encouraged facilities, and HCPs, to adjust behaviour to meet ambitious targets. This exposed HCPs to positive outcomes, with healthy HIV-positive women giving birth to HIV-negative babies. In turn, these successes gradually shifted provider opinions and behaviour regarding safer conception provision [48]. We recommend that high levels of accountability, with measurable targets, be used to drive safer conception care provision, creating further opportunities for HCP exposure to positive experiences. In combination with ongoing eMTCT and UTT achievements, these shifts might further empower HCPs to become change agents themselves, operating within evermore supportive environments, driving widespread, sustainable shifts in attitudes and behaviour.

### Healthcare provider training informed by policy-linked guidelines

Healthcare providers are pivotal to successful safer conception service provision. Two realities undermine South Africa’s progress in this area. First, inclusion of safer conception services in the Contraception and Fertility Planning Policy has not been reflected in provider guidelines or training curricula [51]. Consequently, most providers remain unaware of the current policy and the strategies promoted for HIV-affected couples [16,52,53]. Second, HCPs need comprehensive training to provide client counselling and support to employ safer conception strategies effectively [54]. The lack of training and clinical tools creates HCP uncertainty concerning safer conception strategies, leaving them hesitant to ask clients about their reproductive goals [50,52,55]. In turn, PLHIV avoid discussing their reproductive goals with providers for fear of judgement [14,16,56,57]. It is crucial that comprehensive training materials and clinical tools be developed urgently to enable HCPs to initiate discussions and provide these services.

For providers who are, in principle, supportive of PLHIV having children, uncertainty exists concerning who should take on the task of supporting them: gynaecologists, physicians, nurses or counsellors; within HIV clinics, out-patient departments, family planning services or elsewhere? This perpetuates a practice of “referral as deferral”: clients are referred from counsellor to nurse to doctor to specialist, never accessing the support they need [55,56]. We propose a step-wise training approach to capacitate various providers with different levels of knowledge and skills. First, all primary HCPs, including doctors, nurses and counsellors, require training to routinely screen all reproductive-aged clients, regardless of HIV status, concerning their reproductive goals. Without widespread training and screening, those most at risk of horizontal or vertical HIV transmission may be missed. Then, nurse–clinicians, primarily working within HIV services but also, where possible, reproductive health and family planning services, require more comprehensive, guideline-based safer conception training [1,4] and mentorship to facilitate integrated safer conception service provision. Lastly, a sub-set of public-sector-specialized HIV and gynaecological/fertility doctors should be equipped to manage more complex cases including ART treatment failure or infertility assessments. Accompanying quality improvement activities could further strengthen service quality and sustainability [58].

Training and community mobilization activities are costly. However, considering the numbers who might benefit from safer conception services [6,11,42] and projected cost-savings with each horizontal or vertical HIV infection averted [26,32], this is justifiable. Studies suggest that 30–50% of adults living with HIV may desire to have a child in the near future [6,7,59]. Given the magnitude of the epidemic in South Africa and regionally, hundreds of thousands of individuals could potentially benefit from safer conception services. Here again, we recommend that UTT rollout be used as an opportunity to incorporate safer conception training into existing curricula, thus minimizing costs and alerting different HCP cadres to the importance of routine fertility intention screening and provision of appropriate contraceptive or safer conception services. Engaging community health workers during training could also bolster community outreach, generating greater community awareness of safer conception options.

### Engaging the consumer: PLHIV and their partners

Given the high proportion of PLHIV who desire children [7,59], high consumer demand for safer conception services is expected. However, demand may be counterbalanced by low levels of safer conception knowledge and explicit pregnancy planning amongst potential service users [60]. Pre-emptive discussions about safer conception with all clients, including routine fertility intention screening, followed by targeted safer conception counselling for men and women expressing fertility desires, may help counteract low baseline knowledge levels. Provision of relevant health education materials within primary healthcare facilities may support clients to engage with HCPs regarding this topic [61].

Myriad other challenges hinder HIV-affected couples from accessing safer conception information and engaging in care together, including men remaining disengaged from sexual and reproductive health and services [62], non-disclosure of HIV serostatus to sexual partners [63] and disrupted relationships resulting from employment-related migration [64]. Extensive community engagement and HCP training are needed to address these challenges and shift client and HCP attitudes, particularly towards individuals who cannot engage in care with their partner.

Broader structural and social issues, extending beyond the client–clinic interface, also limit safer conception uptake. Two particular challenges require attention. The first is the high rate of unintended pregnancies in South Africa [8,38], where over 50% of pregnancies are unplanned [10,30]. Over a third of women on ART report unplanned, although not necessarily unwanted, pregnancies [8,10,30], some perhaps seeking to avoid stigma when accessing pregnancy-related services [6,8]. Along with unplanned but wanted pregnancies, many pregnancies are also planned [8]. Community engagement efforts are needed to encourage more people to plan their pregnancies, including engaging with safer conception services. Considering high rates of unplanned pregnancies, even those who do not explicitly communicate a desire for children, may benefit from reinforced messaging around consistent condom use until both partners access HIV testing, disclose their HIV status, and any HIV-positive partner is virologically suppressed on ART. The second challenge involves finding and engaging men and women who remain unaware of their HIV status and/or unlinked to ART care. This group may benefit most from safer conception interventions and yet are the most difficult to draw into healthcare facilities. Nevertheless, desire for an HIV-uninfected infant may be an effective strategy to engage this group in care [62]. We recommend the use of community dialogues and social marketing of safer conception services, within a new, expanded paradigm of health-sustaining behaviours and prevention-oriented services to generate greater demand, particularly amongst those who may benefit most from risk reduction strategies because they are not currently engaged in care.

## Conclusion

Four years after South Africa’s ground-breaking inclusion of safer conception strategies into national policy, services remain scarce. With demonstration projects confirming the potential feasibility, acceptability and suitability of primary healthcare-based safer conception services, action is needed to reinvigorate implementation efforts and disseminate lessons learnt across other high HIV-burden, resource constrained settings. We identified four key implementation barriers: poor policy translation, entrenched organizational cultures, inadequate HCP training and insufficient community engagement. We propose the following recommendations to address these barriers. First, safer conception services could be incorporated into high priority UTT and eMTCT implementation and monitoring frameworks, including promoting a reproductive rights-based approach within these programmes. This will create impetus and accountability for providers to implement safer conception services. Second, a step-wise approach to training, including comprehensive training and mentorship for primary healthcare nurses, needs to be developed and merged into existing UTT and eMTCT training curricula. In settings for which universal scale-up is not feasible, we suggest focusing initial efforts on provinces or districts with the highest HIV prevalence, as well as areas with a high total fertility rate. Lastly, extensive community engagement must be undertaken to generate awareness of, and demand for, these services. The rapid uptake of services at the two demonstration projects in Johannesburg suggests that once services are provided and advertised, and the local communities made aware of the possibility of a safe, supported pregnancy, many are keen to take up the service and remain engaged in the service until pregnancy is achieved and they are linked to eMTCT programmes. Perhaps, people with a desire for an HIV-negative child are not currently accessing existing HIV testing and treatment services because these services are not perceived to be meeting their need – a concern for their unborn child. The progressive steps of creating an innovative safer conception policy and demonstrating its feasibility have been made. Now, the work of implementation across South Africa, and further afield, must begin.

## References

[1] Bekker L-G, Black V, Myer L, Cooper D, Mall S, Mnyami C, et al Guideline on safer conception in fertile HIV-infected individuals and couples. SAJHIVMed. 2011;2011:31–42.

[2] Ciaranello A, Matthews LT. Safer conception strategies for HIV-serodiscordant couples: how safe is safe enough? J Infect Dis. 2015;212:1525–28. 26092857 10.1093/infdis/jiv275 PMC4621252

[3] Heffron R, Davies N, Cooke I, Kaida A, Mergler R, van der Poel S, et al A discussion of key values to inform the design and delivery of services for HIV-affected women and couples attempting pregnancy in resource-constrained settings. J Int AIDS Soc. 2015;18:20272. 26643454 10.7448/IAS.18.6.20272 PMC4672397

[4] Department of Health, South Africa National contraception and fertility planning policy and service delivery guidelines. Pretoria: Department of Health; 2012.

[5] Mantell E, Exner T, Cooper D, Bai D, Leu C-S, Hoffman S, et al Pregnancy intent among a sample of recently diagnosed HIV-positive women and men in Cape Town, South Africa, practicing unprotected sex: correlates and implications for action. JAIDS. 2014;67:S202–9. 25436819 10.1097/QAI.0000000000000369 PMC4251915

[6] Cooper D, Moodley J, Zweigenthal V, Bekker L-G, Shah I, Myer L. Fertility intentions and reproductive health care needs of people living with HIV in Cape Town, South Africa: implications for integrating reproductive health and HIV care services. AIDS Behav. 2009;13:38–46. 19343492 10.1007/s10461-009-9550-1

[7] Schwartz SR, Mehta SH, Taha TE, Rees HV, Venter F, Black V High pregnancy intentions and missed opportunities for patient-provider communication about fertility in a South African cohort of HIV-positive women on antiretroviral therapy. AIDS Behav. 2012;16:69–78. 21656145 10.1007/s10461-011-9981-3

[8] Schwartz SR, Rees H, Mehta S, Venter WD, Taha TE, Black V High incidence of unplanned pregnancy after antiretroviral therapy initiation: findings from a prospective cohort study in South Africa. PLoS One. 2012;7:e36039. 22558319 10.1371/journal.pone.0036039 PMC3338622

[9] Rao A, Baral S, Phaswana-Mafuya N, Lambert A, Kose Z, Mcingana M, et al Pregnancy intentions and safer pregnancy knowledge among female sex workers in Port Elizabeth, South Africa. Obstet Gynecol. 2016 7;128(1):15–21. 27275799 10.1097/AOG.0000000000001471

[10] Myer L, Carter R, Katyal M, Toro P, El-Sadr WM, Abrams EJ Impact of antiretroviral therapy on incidence of pregnancy among HIV-infected women in sub-Saharan Africa: a cohort study. Plos Med. 2010;7:e1000229. 20161723 10.1371/journal.pmed.1000229 PMC2817715

[11] Dinh T, Delaney KP, Goga A Impact of maternal HIV seroconversion during pregnancy on early mother to child transmission of HIV (MTCT) measured at 4–8 weeks postpartum in South Africa 2011–2012: a national population-based evaluation. PLoS One. 2015;10(5):e0125525. 10.1371/journal.pone.0125525 PMC4420458 25942423

[12] Crankshaw T, Smit J, Beksinska ME Placing contraception at the centre of the HIV prevention agenda. Afr J AIDS Res. 2016;15:157–62. 27399045 10.2989/16085906.2016.1204330

[13] Schwartz S, Bassett J, Sanne I, Phofa R, Yende N, Van Rie A Implementation of a safer conception service for HIV-affected couples in South Africa. AIDS. 2014;28:S277–85. 24991901 10.1097/QAD.0000000000000330 PMC4882476

[14] Matthews LT, Crankshaw T, Giddy J, Kaida A, Smit J, Ware N, et al Reproductive decision-making and periconception practices among HIV-positive men and women attending HIV services in Durban, South Africa. AIDS Behav. 2013;17:461–70. 22038045 10.1007/s10461-011-0068-y PMC3560938

[15] Davies N, Mullick S, Schwartz S Uptake and clinical outcomes from a primary healthcare based safer conception service in Johannesburg, South Africa: findings at 7 months. J Int AIDS Soc. 2016;19 Suppl 5:167 Abstract Number: THPDC0105.

[16] Crankshaw T, Mindry D, Munthree C, Letsoalo T, Maharaj P Challenges with couples, serodiscordance and HIV disclosure: healthcare provider perspectives on delivering safer conception services for HIV-affected couples, South Africa. J Int AIDS Soc. 2014;17:18832. 24629843 10.7448/IAS.17.1.18832 PMC3956311

[17] Department of Health, South Africa National consolidated guidelines for the prevention of mother-to-child transmission of HIV (PMTCT) and the management of HIV in children, adolescents and adults. Health NDo, editor Pretoria: National Department of Health; 2015.

[18] Vernazza PL, Graf I, Sonnenberg-Schwan U, Geit M, Meurer A Preexposure prophylaxis and timed intercourse for HIV-discordant couples willing to conceive a child. AIDS. 2011;25:2005–8. 21716070 10.1097/QAD.0b013e32834a36d0

[19] Barreiro P, Del Romero J, Leal M, Hernando V, Asencio R, de Mendoza C, et al Natural pregnancies in HIV-serodiscordant couples receiving successful antiretroviral therapy. J Acquir Immune Defic Syndr. 2006;43:324–6. 17003695 10.1097/01.qai.0000243091.40490.fd

[20] Center for Disease Control and Prevention (CDC) Preexposure prophylaxis for the prevention of HIV infection in the United States – 2014 clinical providers’ supplement. Atlanta, USA: CDC; 2014 p. 1–43.

[21] Schwartz S, Phofa R, Yende N, Bassett J, Sanne I, Van Rie A Clinical outcomes and lessons learned from a safer conception clinic for HIV-affected couples trying to conceive. J Int AIDS Soc. 2016;19 Suppl 5:166. Abstract Number: THPDC0104.

[22] USAID The art of moving from policy to action: lessons learned from the USAID health policy initiative (2005–2010). Washington (DC): USAID; 2010 p. 2.

[23] World Health Organization Guideline on when to start antiretroviral therapy and on preexposure prophylaxis for HIV guidelines. Geneva: World Health Organization; 2015. 26598776

[24] Department of Health, South Africa Implementation of the universal test and treat strategy for HIV positive patients and differentiated care for stable patients. Pretoria: National Department of Health; 2016.

[25] Department of Health, South Africa National policy on HIV pre-exposure prophylaxis (PrEP) and test and treat (T&T). Pretoria: Department of Health; 2016 p. 1–8.

[26] Walensky RP, Ross EL, Kumarasamy N, Wood R, Noubary F, Paltiel D, et al Cost-effectiveness of HIV treatment as prevention in serodiscordant couples. N Engl J Med. 2013;369:1715–25. 24171517 10.1056/NEJMsa1214720 PMC3913536

[27] UNAIDS Start free, stay free, AIDS free. Geneva: UNAIDS; 2016.

[28] World Health Organization Guideline on when to start antiretroviral therapy and on pre-exposure prophylaxis for HIV. Geneva: World Health Organization; 2015. 26598776

[29] Health-e news South Africa moves to ‘test and treat’. Cape Town, South Africa: Health-e news; 2016.

[30] Wilcher R, Petruney T, Cates W The role of family planning in elimination of new pediatric HIV infection. Curr Opin HIV AIDS. 2013;8:490–7. 23743790 10.1097/COH.0b013e3283632bd7 PMC4052828

[31] Van den Berg W, Brittain K, Mercer G, Peakock D, Stinson K, Janson H, et al Improving men’s participation in preventing mother-to-child transmission of HIV as a maternal, neonatal, and child health priority in South Africa. PLoS Med. 2015;12:e1001811. 25849433 10.1371/journal.pmed.1001811 PMC4388663

[32] Goga A, Dinh T-H, Jackson DJ, Lombard C, Delaney KP, Puren A, et al First population-level effectiveness evaluation of a national programme to prevent HIV transmission from mother to child, South Africa. J Epidemiol Commun Health. 2015;69:240–8. 10.1136/jech-2014-204535 PMC4345523 25371480

[33] Bhardwaj S, Barron P, Pillay Y, Treger-Slavin L, Robinson P, Goga A, et al Elimination of mother-to-child transmission of HIV in South Africa: rapid scale-up using quality improvement. Samj. 2014;104:239–43. 24893500 10.7196/samj.7605

[34] McNairy M, Teasdale CA, El-Sadr WM, Mave V, Abrams EJ Mother and child both matter: reconceptualizing the prevention of mother-to-child transmission care continuum. Curr Opin HIV AIDS. 2015;10:403–10. 26352391 10.1097/COH.0000000000000199 PMC4707659

[35] Petruney T, Robinson E, Reynolds H, Wilcher R, Cates W Contraception is the best kept secret for prevention of mother-to-child HIV transmission. Bull World Health Organ. 2008;86:B. 10.2471/BLT.08.051458 PMC2647467 18568260

[36] Mindry D, Milford C, Greener L, Greener RM, Maharaj P, Letsoalo T, et al Client and provider knowledge and views on safer conception for people living with HIV (PLHIV). Sex Reprod Healthc. 2016;10:35–40. 27938871 10.1016/j.srhc.2016.03.005 PMC5155034

[37] Parmelli E, Flodgren G, Beye F, Baillie N, Schaafsma ME, Eccles MP The effectiveness of strategies to change organisational culture to improve healthcare performance: a systematic review. Implement Sci. 2011;6:33. 21457579 10.1186/1748-5908-6-33 PMC3080823

[38] Cooper D, Morroni C, Orner P, Moodley J, Harries J, Cullingworth L, et al Ten years of democracy in South Africa: documenting transformation in reproductive health policy and status. Reprod Health Matt. 2004;12:70–85. 10.1016/s0968-8080(04)24143-x 15626198

[39] Shelton J The provider perspective: human after all. Int Fam Plan Perspect. 2001;27:152–61.

[40] Hill T How clinicians make (or avoid) moral judgments of patients: implications of the evidence for relationships and research. Philos Ethics Humanit Med. 2010;5:11. 20618947 10.1186/1747-5341-5-11 PMC2914676

[41] Walker L, Gilson L ‘We are bitter but we are satisfied’: nurses as street-level bureaucrats in South Africa. Soc Sci Med. 2004;59:1251–61. 15210096 10.1016/j.socscimed.2003.12.020

[42] Beyeza-Kashesya J, Ekstrom AM, Kaharuza F, Mirembe F, Neema S, Kulane A My partner wants a child: a cross-sectional study of the determinants of the desire for children among mutually disclosed sero-discordant couples receiving care in Uganda. BMC Public Health 2010;10. 10.1186/1471-2458-10-247 PMC2877675 20465794

[43] Agadjanian V, Hayford SR PMTCT, HAART, and childbearing in Mozambique: an institutional perspective. AIDS Behav. 2009;13:S103–12. 10.1007/s10461-009-9535-0 PMC2836932 19326206

[44] Wagner G, Linnemayr S, Kityo C, Mugyenyi P Factors associated with intention to conceive and its communication to providers among HIV clients in Uganda. Matern Child Health J. 2012;16:510–8. 21359828 10.1007/s10995-011-0761-5

[45] Goggin K, Mindry D, Beyeza-Kashesyad J, Finocchario-Kesslere S, Wanyenzef R, Nabiryog C, et al “Our hands are tied up”: current state of safer conception services suggests the need for an integrated care model. Health Care Women Int. 2014;35:990–1009. 24901882 10.1080/07399332.2014.920023 PMC4150838

[46] Barroso C, Sippel S Sexual and reproductive health and rights: integration as a holistic and rights-based response to HIV/AIDS. Women’s Health Issues. 2011;21:S250–4. 22055675 10.1016/j.whi.2011.07.002

[47] Baryamutuma R, Baingana F Sexual, reproductive health needs and rights of young people with perinatally acquired HIV in Uganda. Afr Health Sci. 2011;11:211–8. 21857852 PMC3158520

[48] Matthews L, Bajunirwe F, Kastner J, Sanyu N, Akatukwasa C, Ng C, et al “I always worry about what might happen ahead”: implementing safer conception services in the current environment of reproductive counseling for HIV-affected men and women in Uganda. Biomed Res Int. 2016;2016:1–9. 10.1155/2016/4195762 PMC4802028 27051664

[49] Goggin K, Finocchario-Kessler S, Staggs V, Woldetsadik MA, Wanyenze RK, Beyeza-Kashesya J, et al Attitudes, knowledge, and correlates of self-efficacy for the provision of safer conception counseling among Ugandan HIV providers. AIDS Patient Care STDS. 2015;29:1–3. 26588429 10.1089/apc.2015.0089 PMC4684655

[50] Moodley J, Cooper D, Mantell JE, Stern E Health care provider perspectives on pregnancy and parenting in HIV-positive individuals in South Africa. BMC Health Serv Res. 2014;14:384. 25212461 10.1186/1472-6963-14-384 PMC4167138

[51] Department of Health, South Africa National contraception clinical guidelines: a companion to the national contraception and fertility planning policy and service delivery guidelines. Pretoria: Department of Health; 2012.

[52] O’Neil KFM, Greener R, Mosery FN, Safren S, Psaros C, Wilson IB, et al Knowledge, attitudes and practices of safer conception counseling among providers in Durban, South Africa. In: Abstract Book: AIDS 2016, 21st International AIDS Conference, Durban, South Africa, July 18-22, 2016. Geneva, Switzerland: International AIDS Society. Abstract Number: WEPEB120. p. 347.

[53] Matthews L, Milford C, Kaida A, Ehrlich MJ, Ng C, Greener R, et al Lost opportunities to reduce periconception HIV transmission: safer conception counseling by South African providers addresses perinatal but not sexual HIV transmission. J Acquir Immune Defic Syndr. 2014;67:S210–7. 25436820 10.1097/QAI.0000000000000374 PMC4251914

[54] Schwartz SR, West N, Phofa R, Yende N, Sanne I, Bassett J, et al Acceptability and preferences for safer conception HIV prevention strategies: a qualitative study. Int J STD AIDS. 2015;0(0):1–9. 10.1177/0956462415604091 PMC4889551 26384950

[55] West N, Schwartz S, Phofa R, Yende N, Bassett J, Sanne I, et al “I don’t know if this is right … but this is what I’m offering”: healthcare provider knowledge, practice, and attitudes towards safer conception for HIV-affected couples in the context of Southern African guidelines. AIDS Care. 2016;28:390–6. 26445035 10.1080/09540121.2015.1093596 PMC4882088

[56] Matthews LT, Moore L, Milford C, Greener R, Mosery FN, Rifkin R, et al “If I don’t use a condom I would be stressed in my heart that I’ve done something wrong”: routine prevention messages preclude safer conception counseling for HIV-infected men and women in South Africa. AIDS Behav. 2015;19:1666–75. 25711300 10.1007/s10461-015-1026-x PMC4550563

[57] Steiner RJ, Black V, Rees H, Schwartz SR Low receipt and uptake of safer conception messages in routine HIV care: findings from a prospective cohort of women living with HIV in South Africa. J Acquir Immune Defic Syndr. 2016;72:105–13. 26855247 10.1097/QAI.0000000000000945

[58] Umar N, Litaker D, Darcey T Towards more sustainable health care quality improvement in developing countries: the “little steps” approach. Qual Manage Health Care. 2009;18:295–304. 10.1097/QMH.0b013e3181bee28d 19851237

[59] Kaida A, Laher F, Strathdee SA, Janssen PA, Money D, Hogg RS, et al Childbearing intentions of HIV-positive women of reproductive age in Soweto, South Africa: the influence of expanding access to HAART in an HIV hyperendemic setting. Am J Public Health. 2011;101:350–8. 20403884 10.2105/AJPH.2009.177469 PMC3020203

[60] Taylor T, Mantell JE, Nywagi N, Cishe N, Cooper D He lacks his fatherhood: safer conception technologies and the biological imperative for fatherhood among recently-diagnosed Xhosa-speaking men living with HIV in South Africa. Cult Health Sex. 2013;15:1101–14. 23862770 10.1080/13691058.2013.809147 PMC4171952

[61] Coulter AE, Ellins J Effectiveness of strategies for informing, educating, and involving patients. BMJ. 2007;335:24–7. 17615222 10.1136/bmj.39246.581169.80 PMC1910640

[62] Brittain K, Giddy J, Myer L, Cooper D, Harries J, Stinson K Pregnant women’s experiences of male partner involvement in the context of prevention of mother-to-child transmission in Khayelitsha, South Africa. AIDS Care. 2015;27:1020–4. 25738960 10.1080/09540121.2015.1018862

[63] Cooper D, Mantell JE, Nywagi N, Cishe N, Austin-Evelyn K Narrative methods and socio-cultural linguistic approaches in facilitating in depth understanding of HIV disclosure in a cohort of women and men in Cape Town, South Africa. Front Public Health. 2016;4:95. 27242987 10.3389/fpubh.2016.00095 PMC4869124

[64] Cooper D, Moore E, Mantell J Renegotiating intimate relationships with men: how HIV shapes attitudes and experiences of marriage for South African women living with HIV: ‘now in my life, everything I do, looking at my health’. Acta Juridica. 2013;2013:218–38. 25505803 PMC4260330

